# Reward activity in ventral pallidum tracks satiety-sensitive preference and drives choice behavior

**DOI:** 10.1126/sciadv.abc9321

**Published:** 2020-11-04

**Authors:** David J. Ottenheimer, Karen Wang, Xiao Tong, Kurt M. Fraser, Jocelyn M. Richard, Patricia H. Janak

**Affiliations:** 1 The Solomon H. Snyder Department of Neuroscience, Johns Hopkins University, Baltimore, MD, USA.; 2 Department of Psychological and Brain Sciences, Johns Hopkins University, Baltimore, MD, USA.; 3 Department of Neuroscience, University of Minnesota, Minneapolis, MN, USA.; 4 Kavli Neuroscience Discovery Institute, Johns Hopkins University, Baltimore, MD, USA.

## Abstract

A key function of the nervous system is producing adaptive behavior across changing conditions, like physiological state. Although states like thirst and hunger are known to impact decision-making, the neurobiology of this phenomenon has been studied minimally. Here, we tracked evolving preference for sucrose and water as rats proceeded from a thirsty to sated state. As rats shifted from water choices to sucrose choices across the session, the activity of a majority of neurons in the ventral pallidum, a region crucial for reward-related behaviors, closely matched the evolving behavioral preference. The timing of this signal followed the pattern of a reward prediction error, occurring at the cue or the reward depending on when reward identity was revealed. Additionally, optogenetic stimulation of ventral pallidum neurons at the time of reward was able to reverse behavioral preference. Our results suggest that ventral pallidum neurons guide reward-related decisions across changing physiological states.

## Teaser

The brain’s ventral pallidum encodes reward preference across changing physiological state and influences reward decisions.

## INTRODUCTION

Individuals frequently adjust their decision-making across dynamic states, both internal and external. Changes in external states, like the probability of a certain action leading to reward, have been well modeled in the laboratory and have provided key insights into neural signals underlying decisions across dynamic conditions (*1*–*3*). Less is known about how changes in internal states, like hunger or thirst, affect functioning of decision-related neural circuits. Internal states are fundamental to the concept of reward; reward acquisition is strongly motivated by homeostatic drive, and the perceived pleasantness of a reward depends on internal satiety signals (“alliesthesia”) (*4*–*6*). A growing body of work has demonstrated that the reward-related activity of individual neurons across the brain is altered as an animal reaches satiety, a state often accompanied by reduced participation in the experimental task (*7*–*14*). A crucial question remaining is how satiety affects decision-related neural activity when subjects’ preferences are altered by physiological state and the subjects remain engaged in reward-seeking behavior. This topic is critical for understanding how the brain flexibly drives our consumption-related choices.

One brain area potentially important for satiety-sensitive reward processing is the ventral pallidum (VP). This ventral basal ganglia region is hypothesized to integrate information about available rewards to direct reward-related behaviors (*15*, *16*). Reward signals in VP reflect reward preference (*17*–*20*) and are sensitive to physiological state (*13*, *14*, *21*, *22*). In particular, VP neural responses to salt and salt-predicting cues are enhanced by experimentally-induced salt deprivation, while neural responses to sucrose remain stable (*21*, *22*). Along with the orbitofrontal cortex and hypothalamus (*23*–*25*), this makes VP one of the few regions with reports of neural activity that are selectively altered for reward outcomes differentially affected by the physiological state. However, because none of these prior studies presented subjects with a choice between reward outcomes, it is unclear how neural activity in VP (and elsewhere) tracks behavioral preference as physiological state evolves. Moreover, a causal link between VP activity and subjects’ reward choices remains untested. To address these questions, we performed a series of experiments where we recorded from and manipulated VP activity while rats chose between dynamically preferred rewards. We found that the reward activity of a large subset of VP neurons closely matched evolving behavioral preference. Furthermore, optogenetic stimulation of VP at the time of reward biased future choices toward that option. These results establish that VP activity tracks dynamic reward preference at the single neuron level and influences reward decisions.

## RESULTS

### Dynamic preference driven by physiological state

**Fig. 1:**
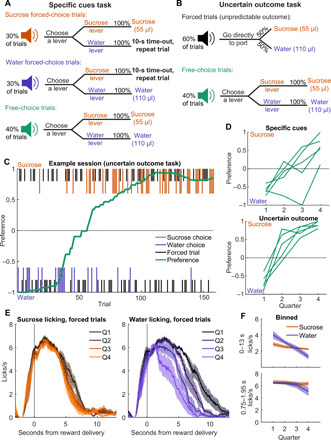
Dynamic preference driven by physiological state. (**A**) Schematic of the “specific cues” task, where there were three trial types, each with a unique auditory cue. Correct lever presses on forced-choice trials led to delivery of the associated reward, while rats could choose to receive water or sucrose on free-choice trials. (**B**) Schematic of the “uncertain outcome” task. The choice trials (and cue) were the same as for the specific cues task, but the forced trials had a different auditory cue and required entry into the reward port rather than a lever press, after which either reward was delivered. (**C**) Example uncertain outcome session, depicting choice trials (colored, longer lines) and forced trials (black, shorter lines) for sucrose (top) and water (bottom), overlaid with preference (green). (**D**) Preference in each task for each of the five rats across four quarters of completed trials. (**E**) Mean (±SEM) lick rate relative to reward delivery across the four quarters (Q) of trials, split into forced sucrose (left) and water (right) trials. Sessions from both tasks are combined here. (**F**) Mean (±SEM) lick rate across 13 s, capturing nearly all of the reward-related licking (top), and within the bin used for neural analysis (0.75 to 1.95 s after delivery).

To model dynamic preference, we designed two tasks where thirsty rats earned either a 55-μl sucrose reward or a 110-μl water reward (Fig. 1). The choice component of both tasks was the same: On 40% of trials, a “choice” auditory cue indicated that rats could press either of the available levers, triggering delivery of the associated reward into the reward port 2 s later. These trials allowed us to assess the rats’ preference for sucrose versus water across the session (Fig. 1, C and D). In the “specific cues” task (Fig. 1A), the remaining 60% of trials were either forced sucrose or forced water trials, each indicated with a distinct auditory cue and requiring the rat to press the correct associated lever, triggering delivery of that reward 2 s later. Because the outcome on forced trials was indicated by the cue, we could evaluate how cue-evoked behavior and neural activity evolved as rats’ preferences shifted. In the “uncertain outcome” task (Fig. 1B), the remaining 60% of trials were forced trials where the rats responded to a single auditory cue by going directly to the reward port, which triggered delivery of either sucrose or water 2 s later with 50/50 probability. By revealing the outcome at either the cue (specific cues task) or reward delivery (uncertain outcome task), we could observe how closely the recorded neural activity followed the pattern of a reward prediction error (*26*), as has been proposed for VP reward-related activity (*14*, *27*–*30*).

In the sessions included here, rats (*n* = 5) completed 88 to 149 (median, 99) trials of the specific cues task and 126 to 175 trials (median, 157) of the uncertain outcome task. In both tasks, rats demonstrated dynamic preference, initially preferring water when thirsty at the beginning of the session and switching to preferring sucrose by the end of the session (Fig. 1, C and D). This was largely driven by a reduced motivation to consume water, evident in maintained licking for sucrose across the session but consistently decreasing licking for water (Fig. 1, E and F). Thus, we succeeded in training rats on a task where, despite unchanging task conditions, the rats demonstrated a preference that shifted according to physiological state, allowing characterization of VP encoding of internally driven changes in preference.

### Dynamic reward encoding in VP occurs when outcome identity is revealed

**Fig. 2:**
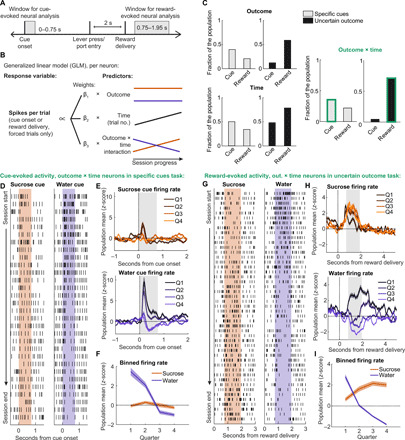
Dynamic reward encoding occurs when outcome identity is revealed. (**A**) Windows used for neural analysis relative to task events. (**B**) Schematic of the GLM used to predict individual neuron activity at time of cue and reward. (**C**) Proportion of neurons from each task with significant impacts of outcome, time, and outcome × time at the time of cue and time of reward. We focused our additional analysis on the outcome × time neurons at time of cue for the specific cues task and at the time of reward for the uncertain outcome task (marked in green outlines). (**D**) Raster from an example outcome × time neuron from the specific cues task, aligned to sucrose (left) and water (right) forced trial cues. Shading indicates window for neural analysis. (**E**) Mean (±SEM) sucrose (top) and water (bottom) cue-evoked firing for all outcome × time neurons from the specific cues task across the four quarters of trials. Gray shading indicates window for neural analysis. (**F**) Mean (±SEM) binned firing for these neurons in this window. (**G**) Raster from example outcome × time neuron from the uncertain outcome task, aligned to sucrose (left) and water (right) delivery on forced trials. Shading indicates window for neural analysis. (**H**) Mean (±SEM) sucrose-evoked (left) and water-evoked (right) firing for all outcome × time neurons from the uncertain outcome task across the four quarters of trials. Gray shading indicates window for neural analysis. (**I**) Mean (±SEM) binned firing for these neurons in this window.

We recorded the activity of individual neurons (single units) from VP while rats performed the specific cues (*n* = 164 neurons) and the uncertain outcome (*n* = 210 neurons) tasks (fig. S1). Because we observed a change in behavioral preference within each session, our goal was to evaluate whether cue- and reward-evoked VP activity also changed across the session and, if so, whether these changes were reward specific. To accomplish this analysis, we implemented a generalized linear model (GLM) that assessed the impact of outcome, time (in numbers of trials), and the interaction between these two predictors (“outcome × time”) on the activity of individual neurons on forced trials at the time of cue or reward (Fig. 2, A and B). We then asked how many neurons from each task were significantly modulated by each predictor (Fig. 2C). We were especially interested in neurons whose activity was predicted by outcome × time and had more positive slopes for sucrose than for water, that is, neurons whose activity tended to increase for sucrose relative to water as the session progressed. We found that the within-trial timing of activity predicted by outcome × time was highly dependent on the task. In the task with specific cues, 35% of neurons had cue-evoked activity that followed this pattern and 22% had reward-evoked activity with this pattern. In the task with uncertain outcome, 4% of neurons had cue-evoked activity with this pattern (essentially noise, because there was only a single, nonspecific cue), and 71% had reward-evoked activity with this pattern. These proportions follow a reward prediction error framework, where outcome-specific responses are encoded by the earliest predictive stimulus; specific cues increased the number of VP neurons with outcome × time activity at the time of cue (*P* < 1 × 10^−14^, χ^2^ test) and decreased the number of neurons with outcome × time activity at the time of reward (*P* < 1 × 10^−20^, χ^2^ test) compared with the task with uncertain outcome. This pattern was present in all five rats (fig. S2).

Our next step was to characterize this evolving cue- and reward-evoked firing in the specific cues and uncertain outcome tasks, respectively. We first examined the activity of the cue-evoked outcome × time neurons (*n* = 58) in the specific cues task. The GLM described a sizable amount of the variance of these neurons’ cue-evoked activity (median *R*^2^ = 0.3). These cue outcome × time neurons were notable for their pronounced excitations to the water cue at the beginning of the session, which decreased and eventually became inhibitions by the end of the session (Fig. 2, D to F). On the other hand, their sucrose cue-evoked activity remained stable across the session (Fig. 2, D to F). This pattern was present on the very first water trial and sucrose trial of the session, so it was not dependent on experiencing the cue-reward pairing in the current physiological state (fig. S3) (*22*, *31*). The ranking of outcome × time neuron activity evoked by each cue switched midway through the session, echoing the switch in behavioral preference (Fig. 1D). These data are noteworthy for demonstrating that individual VP neurons’ cue-evoked representations not only are dependent on physiological state (*13*, *14*) but also are specific to the cue’s associated reward (and the impact of physiological state on that reward), a phenomenon previously seen at a population level across days (*22*) but not on a per-neuron basis as physiological state changes within session. We further found that the cue-evoked activity of outcome × time neurons predicted the latency to press the lever on a trial-by-trial basis (fig. S4), suggesting that these VP cue representations could invigorate reward-seeking actions. The remaining, non–outcome × time neurons had weaker correlations with lever pressing (fig. S4F) and minimal modulation around cue onset (fig. S5, A and B).

We next examined the reward-evoked activity of outcome × time neurons (*n* = 149) in the uncertain outcome task. Impressively, the GLM described a large amount of the variance of these neurons’ reward-evoked activity (median *R*^2^ = 0.47). Working within a prediction error framework, reward delivery resolves the uncertainty of which reward will be delivered; therefore, this signal should reflect a positive error when receiving the preferred reward and a negative error when receiving the nonpreferred reward. We thus were interested to determine whether water-evoked activity decreased across the session as it became less preferred, and sucrose-evoked activity increased. In contrast to specific cues cue-evoked activity, which decreased for both cues across the session (Fig. 2, D to F), this was indeed the case for the reward-evoked activity of outcome × time neurons, which had decreasing activity for water and increasing activity for sucrose (Fig. 2, G to I). Again, the ranking of the activity of these outcome × time neurons for the respective rewards switched during the session (Fig. 2I), mirroring the switch in behavioral preference from these uncertain outcome sessions (Fig. 1D). The remaining, non–outcome × time neurons from the uncertain outcome task had little modulation around reward delivery (fig. S5, C and D).

### VP reward-evoked activity accurately predicts behavioral preference

**Fig. 3:**
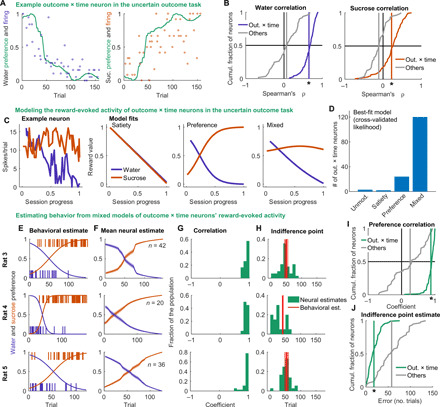
Reward-evoked activity accurately predicts behavioral preference. (**A**) Normalized reward-evoked firing of an example outcome × time neuron in the uncertain outcome task on forced trials overlaid with preference for water (left) and sucrose (right). (**B**) Distribution of correlation coefficients between firing rate and preference for outcome × time (blue or orange) and non–outcome × time (gray) neurons on forced water trials (left) or forced sucrose trials (right). Vertical line is mean. *P < 1 × 10^−24^, Wilcoxon rank sum test comparing outcome × time and non–outcome × time neurons on water trials, and *P* < 1 × 10^−13^ for sucrose trials. (**C**) Example fits of three models we considered to describe the activity of outcome × time neurons in the uncertain outcome task at time of reward delivery: satiety, preference, and mixed, which linearly combined satiety and preference. (**D**) Distribution of best-fit model for all outcome × time neurons, determined with cross-validated likelihood. (**E**) From three example sessions, the choices of the rats across the session and the preference estimated with a logistic function. (**F**) Mean (±SEM) estimate of preference from fits of the mixed model to the outcome × time neurons from these sessions. (**G**) Correlation between neural estimate and behavioral estimate of preference for each outcome × time neuron. (**H**) Estimates of the indifference point (sucrose and water equally preferred) from the neural and behavioral (±SE) models. (**I**) Across all uncertain outcome sessions, outcome × time neurons had preference estimates with higher correlations with the behavioral estimate than the remaining non–outcome × time neurons did (*P* < 1 × 10^−21^, Wilcoxon rank sum test). (**J**) Outcome × time neurons’ estimates of indifference point were closer to the behavioral estimate than the remaining non–outcome × time neurons (*P* < 1 × 10^−15^, Wilcoxon rank sum test).

The particularly strong dynamic reward-specific activity at the time of reward in the uncertain outcome task, with decreasing activity for water and increasing activity for sucrose, encouraged us to explore how this signal related to rats’ choice behavior across the session, which also followed this general pattern. First, we compared the reward-evoked activity of outcome × time neurons on forced water and sucrose trials with the choice-derived preference for the respective reward. We found a strong correlation between the two, particularly in comparison with the weaker correlation for non–outcome × time neurons (Fig. 3, A and B). Nevertheless, we noticed that, on average, the neural responses of outcome × time neurons to sucrose and water do not change symmetrically across the session (Fig. 2I), as might be expected for an error signal driven purely by preference. Because the overall value of the task declines as the rats become sated, we hypothesized that the outcome × time reward-evoked activity in VP may reflect a prediction error where the value of the outcome is derived from a combination of satiety and preference (because there is an equal probability of receiving sucrose and water on these trials, the prediction is the same regardless of outcome, and the prediction error will be proportional to the value of the outcome). To evaluate how well satiety and preference can explain the activity of VP outcome × time neurons, we designed a series of models incorporating these features that could be fit to the activity of individual outcome × time neurons (Fig. 3C). The first, “unmodulated,” has no reward-specific or satiety-related modulation. The second, “satiety,” is a linear approximation of declining motivation that decreases uniformly with each trial. The third, “preference,” is a logistic function with midpoint and steepness as free parameters to flexibly capture the continuous, opposing changes in preference for each reward across the session. The final, “mixed,” is a linear combination of Satiety and Preference with an additional free parameter determining their relative weights. We fit all four models to the reward-evoked activity of outcome × time neurons in the uncertain outcome task with maximum likelihood estimation and determined which model best described each neuron’s activity with cross-validated likelihood. The mixed model best described the activity of the vast majority of outcome × time cells (Fig. 3D), suggesting that the activity of these neurons is influenced by both preference and satiety.

To determine how closely reward-evoked outcome × time neuron activity related to the rats’ choices in the uncertain outcome sessions, we next attempted to predict behavioral preference using the activity of outcome × time cells. During the model fitting process, maximum likelihood estimation finds the values of the model parameters that best describe the activity of each neuron. From the parameters associated with the mixed model, we could estimate the rat’s preference across the session. Because each neuron was fit individually, each neuron produces a prediction of the rat’s preference based on its best-fit parameters. To compare the outcome × time neuron estimates of preference to the rat’s behavioral preference, we fit the same logistic function in the mixed model to the choices from each session (instead of neural activity) and found the best parameters (Fig. 3E). Preference estimates from the outcome × time neurons in each session agreed well with the behavioral estimates (Fig. 3, F and G). The preference estimates from outcome × time neurons were better correlated with the behavioral estimate than the estimates from the remaining neurons (Fig. 3I). The logistic function also explicitly estimates the indifference point, the point at which the preference for the two rewards is ambivalent; there was generally high agreement between neural and behavioral estimates of indifference point among outcome × time neurons (Fig. 3H), which outperformed the remaining neurons (Fig. 3J). These metrics indicate that, despite being derived completely independently of the choice behavior, outcome × time neuronal estimates of preference predicted choice behavior well.

To confirm the robustness of these findings, we performed the same analysis on neurons (*n* = 112) recorded during four additional uncertain outcome sessions from four of the rats. These sessions had fewer neurons, and two did not meet our inclusion criteria, so they provided a strict test of the reproducibility of this method to estimate preference from neural activity. Notably, these neurons had a similarly high proportion of neurons with a significant impact of outcome × time on reward activity (66%). Overall, the reward outcome × time neurons from these additional sessions were also able to accurately predict behavioral preference and did so better than the remaining non–outcome × time neurons (fig. S6). These results indicate that VP reward activity very reliably tracks behavioral preference.

### VP reward-evoked activity instructs choice behavior

**Fig. 4:**
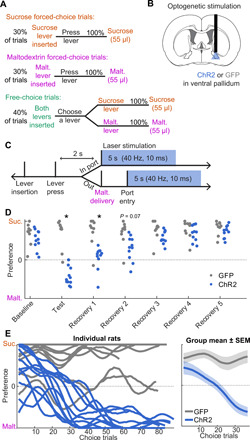
Stimulation of VP at reward delivery biases choice behavior. (**A**) Task design for optogenetic stimulation experiment. Rats chose between sucrose and maltodextrin. Levers were retracted after press. (**B**) Optic fibers and virus containing ChR2 (or GFP control) were implanted/infused bilaterally in VP, but only the right hemisphere was stimulated in this experiment. (**C**) During the test session, on maltodextrin trials, VP was photostimulated unilaterally for 5 s at 40 Hz, beginning with maltodextrin delivery, or whenever the rat first entered the port thereafter, to overlap with maltodextrin consumption. (**D**) Preference for sucrose versus maltodextrin on choice trials at baseline (after training), on test session, and for five recovery days after without laser. There was a significant interaction between day and group across these seven sessions (*F*_6,126_ = 10.6, *P* < 0.00000001). Post hoc Tukey tests (corrected for multiple comparisons) revealed a significant difference between groups on test day (*P* < 0.000001) and the first recovery day (*P* < 0.00001). (**E**) Preference (smoothed) on choice trials across test session for individual rats (left) and averaged for each group (right).

Above, we showed that reward-related activity in VP is closely related to behavioral preference and satiety and that this activity occurs at the moment when the outcome is indicated, resembling a reward prediction error. If VP reward activity serves as a reward prediction error, then this activity would have the ability to inform future preferences by updating the value of the recently chosen option. We next sought to test this hypothesis by manipulating reward-evoked activity in VP directly. Prior work has shown that stimulation of VP is positively reinforcing (fig. S7) (*14*, *32*, *33*), but the role of VP in modifying reward-seeking actions in a decision-making context is unclear. We hypothesized that artificially elevating VP activity following delivery of a less-preferred reward (mimicking the positive prediction error following receipt of the preferred reward in the uncertain outcome task) would bias preference toward that option in the future. To test our hypothesis, we implanted a new group of rats with optic fibers and virus containing channelrhodopsin (*n* = 11; fig. S1) or green fluorescent protein (GFP) control (*n* = 9) and trained them on a modified version of the specific cues task with sucrose and maltodextrin as rewards (Fig. 4, A and B). We chose these rewards because rats consistently prefer sucrose over maltodextrin (*17*, *34*), permitting a more stable backdrop for our manipulation than sucrose and water. After training, we ran a test session where we stimulated VP concurrent with maltodextrin receipt: either the moment when maltodextrin was delivered if rats were in the reward port, or else upon the first port entry following maltodextrin delivery (Fig. 4C). We stimulated at 40 Hz with 10-ms pulse width, parameters previously shown to be maximally reinforcing in VP (*33*). Impressively, pairing maltodextrin with VP stimulation shifted rats’ preference from the sucrose lever to the maltodextrin lever on choice trials (Fig. 4D); this shift could be tracked within the session as the rats experienced additional laser-paired maltodextrin trials (Fig. 4E). In addition, while stimulation had no effect on lever press latency on the subsequent trial, it did bias rats toward quicker presses on maltodextrin trials relative to sucrose trials (fig. S8). Unexpectedly, this shift in maltodextrin lever preference persisted for at least one additional day when laser stimulation was withheld (Fig. 4D), demonstrating that VP activity at the time of reward can induce persistent behavioral preferences.

## DISCUSSION

Our findings here unify and expand upon a number of previous observations of VP activity. Prior work has demonstrated that VP neural activity is sensitive to preference (*17*–*20*) and satiety (*13*, *14*, *21*, *22*), has some features of a reward prediction error (*14*, *27*–*30*), and is reinforcing (*14*, *32*, *33*), although the interaction between these distinct aspects of VP signaling was unclear because they were characterized in separate experiments. Our data also build upon reports of individual neurons in the orbitofrontal cortex and hypothalamus whose responses diminish for rewards (and their predictive cues) fed to satiety but remain intact for other rewards (*23*–*25*). Our work here is an important advance over all these findings because, by implementing a behavioral task that allows rats to choose their preferred reward as they proceed from thirsty to sated, we were able to demonstrate that VP activity was tightly linked not just with physiological state but also with the subjects’ ongoing choices as they were influenced by their physiological state. With the additional result that optogenetic stimulation of VP biased behavioral preference, we propose that reward signaling in VP integrates physiological state and reward preference to direct subjects’ choices. These data lend clarity to the poorly characterized functional role of VP (and other satiety-sensitive reward signals) in decision-making.

These findings are part of a larger body of work characterizing the impact of satiety on brain-wide neural dynamics. There is evidence that, across the brain, task-related activity in individual neurons is reduced when animals are sated, and this reduction in activity is accompanied by less engagement in the behavioral task (*7*–*14*). By presenting subjects with multiple reward outcomes differentially affected by satiety, we were able to measure not just reduced motivation for water but also an increased preference for sucrose as rats became less thirsty. This allowed us to observe an enhancement in sucrose-related activity within a single uncertain outcome session, in contrast to the predominant finding from previous work that satiety uniformly blunts reward-related activity. Thus, our work establishes the importance (and feasibility) of studying how satiety influences not just overall motivation but also deliberative processes comparing different rewards and physiological needs.

Another notable finding from our work is that, like many observations of dopamine neurons, the timing of preference-sensitive signaling in VP followed the general framework of a reward prediction error (*26*). In particular, there was a notable difference in the number of neurons tracking reward preference at the time of reward delivery in the specific cues and uncertain outcome tasks, in which the reward identity on a given trial is signaled by a preceding cue, or not, respectively. The fact that there were many more neurons with this preference-related activity at the time of reward when the outcome had not already been signaled to the rat suggests that VP encodes an error signal derived from momentary reward preference (if VP merely encoded the preference of the received reward, we would see this signal in both tasks). The interaction between satiety, preference, and reward prediction errors has not been characterized well, so our results are some of the first data showing that the hallmark transfer of error signal from reward to cue (*26*) can occur in scenarios where the value of the outcomes changes (and switches ranking) across the session. This type of signal could not be easily explained in a model-free framework where the value of cues is updated only by the value of the received outcome irrespective of reward identity. This is most clearly seen in the specific cues task where the very first presentation of the water cue evokes very strong firing, indicating a high valuation, despite the low firing and low value of water at the conclusion of the previous session [see also (*22*)]. Thus, in some scenarios, VP encodes a model-based signal that takes into account the current value of the predicted outcome (*35*). This result is also consistent with frameworks incorporating prediction errors and incentive salience (*22*, *31*, *36*–*38*).

Our data indicate that VP is a functionally important node within the brain circuits that process the value of available rewards. How these VP signals interact with a brain-wide network to drive decision-making remains to be determined. Given the reward prediction error-like signaling we observed in VP and the connectivity between VP and the dopamine system (*16*, *28*, *32*, *33*), it will be important to clarify both the influence of these regions on each other and their separable roles in reward prediction error signaling. There have been mixed reports of prediction errors in VP (*14*, *27*–*30*). We previously characterized a robust reward prediction error signal in VP where predictions were derived from the previously received reward outcomes, but we saw less of an influence of predictions derived from reward-specific cues (*29*). An important difference in the current experiment compared with our and others’ prior work is that, not only are the cues specific to the outcome, but they also require the rats to perform distinct actions (pressing the appropriate lever). Moreover, the relative value of the outcomes changes throughout the session, which may make the cue-action-outcome contingencies particularly salient to the rats. Thus, the relatively prominent reward prediction error-like signaling we observed here could indicate that VP is especially recruited to update the value of particular actions in dynamic reward-seeking settings, an idea supported by our finding that optogenetic stimulation of VP biased rats’ reward-seeking actions across a single session. Future work will need to determine whether the complementary experiment, inhibition of VP following delivery of a preferred reward, can bias future choices away from that option.

Another important future direction will be to integrate the current findings with known heterogeneity within VP. There has been considerable work delineating distinct connectivity and functions of VP subregions (*15*, *16*). Nevertheless, consistent with previous in vivo electrophysiology studies (*17*, *38*), we saw little evidence for spatially distinct differences in reward encoding; more systematic and precise approaches are necessary to confirm the presence or absence of regional differences. There is also a growing literature on the roles of different cell types in VP in motivated behavior, especially the opposing roles of glutamatergic and GABAergic populations (*14*, *32*, *33*, *39*). Given the evidence that GABAergic neurons make up the majority of the VP population and that they positively encode value and promote reward-seeking behavior, we speculate that the outcome × time neurons we describe here are GABAergic. Future studies would need to clarify if this is the case and whether our optogenetic findings replicate when targeting GABAergic cells specifically. It will also be important to consider more carefully the link between physiology and optogenetic manipulations; although 40-Hz optogenetic stimulation is maximally reinforcing (*33*), as we use here, this is greater than typically observed in vivo. Precise augmentation or reduction of existing VP signals would provide a more rigorous test for the role of observed neural dynamics in reward-seeking behavior. Overall, these findings reveal that VP encodes reward prediction error-like signals derived from dynamic reward preference and emphasize the importance of a continued, detailed study of VP to understand reward learning and decision-making processes affected by physiological state.

## MATERIALS AND METHODS

### Animals

Subjects were male and female Long-Evans rats from Envigo weighing 200 to 275 g at arrival and single-housed on a 12-hour light/dark cycle. Rats were given free access to food in their home cages for the duration of the experiment and maintained above 90% baseline body weight. For training and test sessions, they were water restricted overnight. All experimental procedures were performed in strict accordance with protocols approved by the Animal Care and Use Committee at the Johns Hopkins University.

### Reward solutions

We used 10% solutions by weight of sucrose (Thermo Fisher Scientific, MA) and maltodextrin (SolCarb, Solace Nutrition, CT) in tap water or tap water alone. Before behavioral training, rats were given 1 day of free access to sucrose and/or maltodextrin solution in their home cages depending on the rewards used for the experiment to permit acclimation to the rewarding solutions.

### Surgical procedures

Rats were anesthetized with isoflurane (5%) and maintained under anesthesia for the duration of the surgery (1 to 2%). Rats received injections of carprofen (5 mg/kg) and cefazolin (70 mg/kg) before incision.

### Electrophysiology

Drivable bundles of 16 tungsten wires were implanted in VP [+0.5 mm anterior-posterior (AP), +2.4 mm mediolateral (ML), and −8 mm dorsoventral (DV)] of trained rats.

### Optogenetics

We infused 0.7 μl of virus containing channelrhodopsin [AAV5-hsyn-hChR2(H134R)-EYFP, 1.7 × 10^13^ viral particles/ml from Addgene, gift from K. Deisseroth] or control virus (AAV5-hsyn-EGFP, 1.2 × 10^13^ viral particles/ml from Addgene, gift from B. Roth) bilaterally in VP (+0.5 mm AP, ±2.5 mm ML, and −8.2 mm DV) at a rate of 0.1 μl/min for 7 min. We then implanted 300-μm-diameter optic fibers 0.3 mm above.

### Histology

Deeply anesthetized rats were perfused intracardially with 0.9% saline followed by 4% paraformaldehyde. Brains were postfixed in 4% paraformaldehyde for 24 hours and then transferred to 25% sucrose for a minimum of 24 hours before being sectioned in 50-μm slices on a cryostat.

### Electrophysiology

Electrode sites were labeled by passing a DC through each electrode before perfusion. Slices were stained with cresyl violet to determine recording sites.

### Optogenetics

Slices were coverslipped with Vectashield mounting medium with 4′,6-diamidino-2-phenylindole (DAPI) and imaged. Viral expression was determined from the fluorescence of the expressed virus.

### Recording and spike sorting

Electrical signals and behavioral events were collected from freely moving rats with OmniPlex (Plexon) as in (*17*, *29*). Waveforms were sorted into units using offline sorter (Plexon), and any units that were not detectable for the entire session were discarded. When isolating units, to ensure that they were single units rather than multi-units, we examined the auto- and cross-correlograms of candidate units, plotted waveform features over time to ensure unimodal continuity, and discarded any units with more than 0.2% of spikes within a 2-ms window of another spike, a conservative estimate of refractory period. The resulting units had baseline firing rates ranging from 0.2 to 9.7 Hz. The 25th, 50th, and 75th percentiles were 3.7, 4.8, and 5.7 Hz, respectively.

### Behavioral tasks (electrophysiology)

The behavioral apparatus consisted of two retractable levers (Med Associates), one on each side of a reward port. Rats were trained to associate each lever with a distinct reward: 55 μl of sucrose or 110 μl of water. The pairing of rewards with levers was counterbalanced across rats but remained the same for each rat across days. Rats were first trained on FR1 with both levers present and then were moved to a mixture of forced- and free-choice trials with lever retracting upon successful press. Once rats were trained, the levers remained extended for the duration of the session. For the specific cues task, trial types (forced sucrose, 30% of trials; forced water, 30% of trials; or choice, 40% of trials) were announced by distinct auditory cues (white noise, pure tones, or siren, assignments counterbalanced). Trials were randomly interspersed throughout the session. If the rat selected the incorrect lever on forced trials, both levers retracted for 10 s, and then the rat could correct its mistake upon reinsertion. Cues remained on until the rat selected a correct lever; correct presses triggered vacuum-mediated evacuation of any residual liquid in the reward cup and delivery of the lever-associated reward 2 s later. There was a 20- to 45-s intertrial interval following reward delivery before the next cue onset. Rats had experienced ≥10 sessions with final contingencies when recording started, and all performed above chance on forced-choice trials (57 to 97% accuracy; median, 65%). After we completed recordings from these sessions, rats were trained on the second task, uncertain outcome. In this task, there were two trial types: forced (60% of trials) and choice (40% of trials). Instead of distinct auditory cues announcing forced sucrose and water trials, a fourth auditory cue (lower-frequency siren) indicated that the rat should go directly to the reward port, which terminated the cue and triggered random delivery of sucrose or water 2 s later. Lever presses had no effect on these trials. Choice trials remained the same as specific cues. Sessions were self-paced; we stopped the session after 90 min. In total, five rats (three males and two females) completed these sessions and had electrodes successfully targeted to VP. We analyzed one session from each rat (for each of the two tasks) that best reflected the average trend across all sessions: water preference in the first quarter of trials, sucrose preference in the final quarter, and relatively monotonic transition. Electrodes remained in the same location for the duration of the experiment.

### Optogenetic manipulations

Rats were connected unilaterally via ceramic mating sleeves to a 200-μm core patch cord, which interfaced with a 473-nm DPSS (diode-pumped solid-state) laser (Opto-Engine LLC). Laser delivery during test sessions was initiated by signals from MedPC SmartCTRL cards to a Master-9 Stimulus Controller (AMPI).

For this experiment, rats were trained on a sucrose/maltodextrin choice task where they earned 55 μl of either reward. Trial frequency was 30% forced sucrose, 30% forced maltodextrin, and 40% choice, randomly interspersed. The available levers extended at trial onset, and all extended levers retracted after a press was made, triggering reward delivery 2 s later. Lever assignments were counterbalanced. Rats received 12 days of training on the final task before testing. On test session, maltodextrin delivery was paired with unilateral 40-Hz pulsed photoexcitation of VP for 5 s (10-ms pulse width, 10 to 12 mW), parameters we selected for being maximally reinforcing (*33*). Although rats were implanted bilaterally, we stimulated the right hemisphere only in all rats for this test for consistency and for a stronger test of sufficiency. We only included rats who had their right fiber and viral expression in VP. This resulted in 11 rats with ChR2 (5 males and 6 females) and 9 rats with GFP (4 males and 5 females). For intracranial self-stimulation, the same rats were given access to two previously occluded nosepoke ports in the same behavioral chambers. Entry into one port triggered 1 s of 40-Hz stimulation (10-ms pulse width, 10 to 12 mW) of the right hemisphere.

### Analyzing trials in session quarters

Because the sessions were self-paced, each rat completed a different number of trials (88 to 149; median, 99 for specific cues; 126 to 175; median, 157 for uncertain outcome). To analyze changes across the session, we elected to group trials into quarters of total completed trials, which should ensure similar levels of motivation (within task), rather than dividing sections by a set number of trials.

### Behavioral analysis

We estimated preference across choice trials by smoothing the rats’ choices (0 for water and 1 for sucrose) with a Gaussian filter (σ = 5). We then estimated preference on forced trials by assigning each forced trial the smoothed preference of the nearest choice trial. Preference within a given quarter was calculated by finding the fraction of choices from that quarter that were sucrose; this was then linearly transformed from −1 to 1. For analysis of lever press latency, we performed a natural log transformation of the time interval between cue onset and first correct lever press. The mean latency and licking per quarter were calculated per session, so each session only contributed 1 point to each quarter.

### PSTH creation

To construct peristimulus time histograms (PSTHs), we used 0.01-ms bins surrounding the event of interest. PSTHs were first smoothed on an individual trial basis using a half-normal filter (σ = 3) that only used activity in previous, but not upcoming, bins. Then, the PSTH across all trials was smoothed with another half-normal filter (σ = 8). Each bin of the PSTH was *z* scored by subtracting the mean firing rate across 10-s windows before each trial and dividing by the SD across those windows. PSTHs for licking were created in the same manner (without *z* scoring) with only one round of smoothing after PSTH creation, σ = 25.

### Generalized linear model

To determine the influence of time and outcome on cue- and reward-evoked firing, we fit a GLM with a Poisson distribution to the unsmoothed, binned activity of each neuron on forced trials (“fitglm” in MATLAB). For cue activity, we used a bin 0 to 0.75 s following cue onset, which captured the majority of the phasic response to the cue (Fig. 2). For reward activity, we used a bin 0.75 to 1.95 s following reward delivery to remain consistent with our previous work (*29*), where we saw this was a bin particularly sensitive to modulation by previous outcome. We only included trials where the rat was in the reward port during reward delivery. For the GLM’s predictors, we used trial number as a proxy for time, the outcome on each trial, and the interaction between these two predictors (outcome × time). Significant predictors were determined by the fitglm function in MATLAB with a cutoff of *P* < 0.05.

### Correlations with latency and preference

We calculated correlations for cue activity with lever press latency and reward activity with preference using the nonparametric Spearman’s ρ. We chose this test because we wanted to assess covariance among the variables of interest without assuming a linear relationship. For cue activity and lever press latency, we included all trials (including choice trials). For reward activity and preference, we only looked at forced trials.

### Model fitting

These methods are adapted from (*29*). For each neuron, we took the spike count, *s*(*t*), within the 0.75- to 1.95-s postreward delivery time bin for each forced trial and fit the following four Poisson spike count models. We only included trials where the rat was in the reward port during reward delivery. For all but the unmodulated model, we used *a* as a slope (gain) and *b* as an intercept (offset) parameter to map the model values to spike counts.

#### Unmodulated model

$$s(t)∼\text{Poisson}(\text{exp}(\text{ln}(\bar{s})))$$

where $\bar{s}$ is the mean firing rate across all trials.

#### Satiety model

$$\begin{array}{c} \text{Sat}(t)=1-\frac{t}{t_{\text{end}}}\\ s(t)∼\text{Poisson}(\text{exp}(a\cdot \text{Sat}(t)+b)) \end{array}$$

where *t*_end_ is the total number of trials.

#### Preference model

$$\begin{array}{c} \text{For sucrose trials}\\ \text{Pref}(t)=\frac{1}{1+e^{-k\cdot (t-t_{0})}}\\ \text{For water trials}\\ \text{Pref}(t)=1-\frac{1}{1+e^{-k\cdot (t-t_{0})}}\\ s(t)∼\text{Poisson}(\text{exp}(a\cdot \text{Pref}(t)+b)) \end{array}$$

where *k* is the steepness of the curve and *t*_0_ is the midpoint (in trials). The convention for the logistic function was to approximate sucrose preference (increasing throughout the session), so it needed to be inverted for water trials. Preference (*Pref*) was then normalized from 0 to 1 across all trials in the session before being transformed into spikes.

#### Mixed model

$$\begin{array}{c} \text{SWP}(t)=w\cdot \text{Pref}(t)+(1-w)\cdot \text{Sat}(t)\\ s(t)∼\text{Poisson}(\text{exp}(a\cdot \text{SWP}(t)+b)) \end{array}$$

where Pref and satiety (*Sat*) are found as above and *w* determines the relative contribution of each to the satiety-weighted preference (*SWP*).

For all models with a slope parameter, we constrained the slope, *a*, to be >0, as our previous work demonstrated that the majority of outcome-selective VP neurons are positively correlated with value. We found maximum likelihood estimates for each model and selected the best model using cross-validated likelihood, which was calculated by finding the mean likelihood from 50 repetitions of fitting the model parameters to 80% of the data and testing these parameters on the remaining 20%. We used 20 randomly selected starting initial values for each parameter to avoid finding local minima.

We also tried a model where we inputted the total number of licks on each trial to predict firing rate, as a possible alternative explanation for the across-session changes in VP activity. When we included this model in the comparison, it was the best model for 10 of 149 reward outcome × time neurons in the uncertain outcome task in contrast to 111 of 149 best fit by the mixed model. Thus, the number of licks was not a better predictor of outcome × time neuron activity than the mixture of preference and satiety.

To find the logistic function estimate of preference from the choice behavior (Fig. 4E), we used “nlinfit” in MATLAB to find the best parameters for fitting the *Pref* equation above to the choices on each trial (rather than neural activity) for a given session. To compare this to the neural estimate of preference from neurons recorded during that session, we used the values of the logistic function parameters from the mixed model, found during the fitting process, to generate a per-neuron estimate of behavioral preference across the session. The *t*_0_ from the mixed model gave us the neural estimates of midpoint.

### Data availability

The data and code used to analyze the data are available on GitHub: https://github.com/djottenheimer/dynamic-preference

## Supplementary Material

http://advances.sciencemag.org/cgi/content/full/6/45/eabc9321/DC1

Adobe PDF - abc9321_SM.pdf

Reward activity in ventral pallidum tracks satiety-sensitive preference and drives choice behavior

## SUPPLEMENTARY MATERIALS

Supplementary material for this article is available at http://advances.sciencemag.org/cgi/content/full/6/45/eabc9321/DC1
