# Supplementary material for: Reward activity in ventral pallidum tracks satiety-sensitive preference and drives choice behavior
Source: Sci Adv. 2020 Nov 4;6(45):eabc9321. doi: 10.1126/sciadv.abc9321 (PMC7673692; doi:10.1126/sciadv.abc9321)
Supplement: http://advances.sciencemag.org/cgi/content/full/6/45/eabc9321/DC1 [file supp_6_45_eabc9321__index.html]

Science Advances | Science AdvancesAAASSearchScience AdvancesMenu

## Supplementary Materials

# Reward activity in ventral pallidum tracks satiety-sensitive preference and drives choice behavior

David J. Ottenheimer, Karen Wang, Xiao Tong, Kurt M. Fraser, Jocelyn M. Richard, Patricia H. Janak

Download Supplement

**This PDF file includes:**

- Figs. S1 to S8

**Files in this Data Supplement:**

- Adobe PDF - abc9321\_SM.pdf
