# Supplementary material for: Reward activity in ventral pallidum tracks satiety-sensitive preference and drives choice behavior
Source: Sci Adv. 2020 Nov 4;6(45):eabc9321. doi: 10.1126/sciadv.abc9321 (PMC7673692; doi:10.1126/sciadv.abc9321)
Supplement: http://advances.sciencemag.org/cgi/content/full/6/45/eabc9321/DC1 [file supp_6_45_eabc9321__1.pdf]

Science Advances
AAAS

advances.sciencemag.org/cgi/content/full/6/45/eabc9321/DC1

Supplementary Materials for

Reward activity in ventral pallidum tracks satiety-sensitive preference and drives choice behavior

David J. Ottenheimer, Karen Wang, Xiao Tong, Kurt M. Fraser, Jocelyn M. Richard, Patricia H. Janak*

*Corresponding author. Email: patricia.janak@jhu.edu

Published 4 November 2020, *Sci. Adv.* **6**, eabc9321 (2020)
DOI: 10.1126/sciadv.abc9321

This PDF file includes:

Figs. S1 to S8

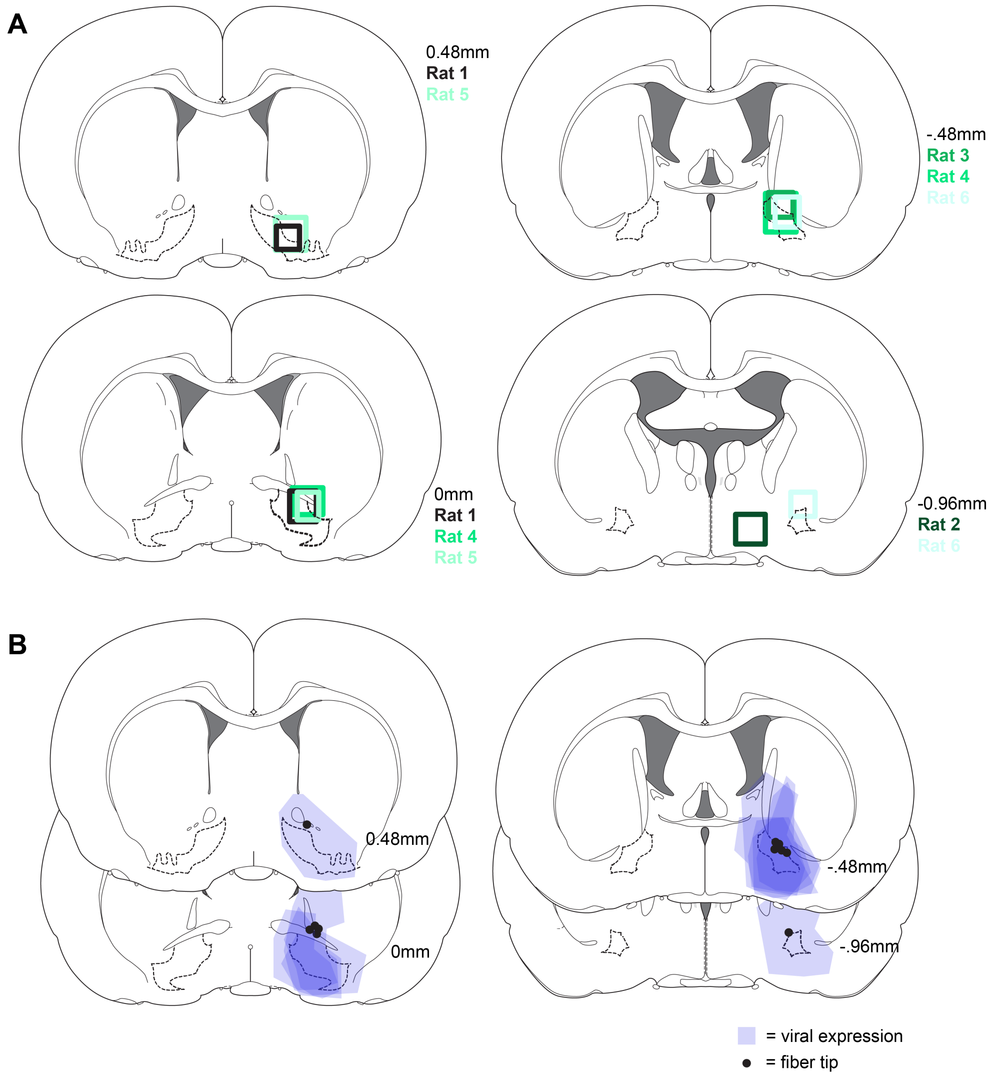

Figure S 1: Placement for electrodes, fibers, and virus.

(A) Placements for electrophysiology recordings. Boxes indicate tissue damage on the specified coronal section. Because wires were maintained in the same location for the duration of the experiments, all recordings were taken at the bottom of the demarcated damage. Rat 2 was excluded from analysis.

(B) Placements for ChR2 experiments.

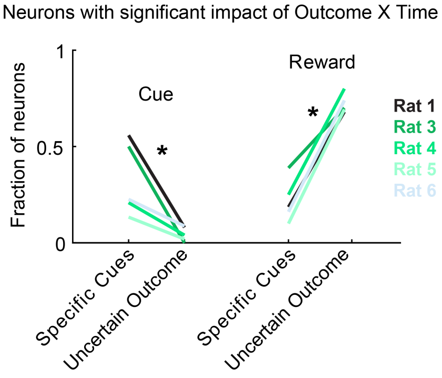

Figure S 2: Outcome X Time neurons in individual rats.

(A) Proportions of neurons with a significant impact of Outcome X Time on cue (left) and reward (right) firing, plotted for individual rats. When considering each rat as one data point,the number of neurons with Outcome X Time modulation at time of the cue was significantly lower ($p = 0.03$, paired t-test) and higher at the time of reward ($p = 0.0006$, paired t-test) in the Uncertain Outcome task compared to the Specific Cues task.

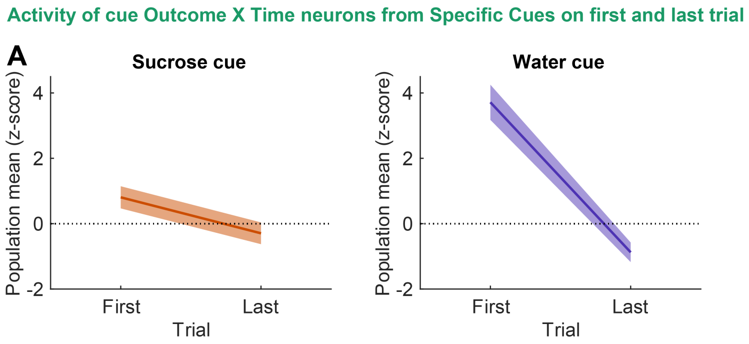

Figure S 3: Water cue evokes robust excitation on first trial of Specific Cues task.

(A) Mean(+/−SEM) firing of Outcome X Time neurons 0-0.75s following sucrose (left) and water (right) cue onset on the first and last trial of each type in the Specific Cues sessions. On the first water cue trial, Outcome X Time neurons had robust excitation, exceeding firing on the first sucrose cue trial ($p = 0.000009$, Wilcoxon signed-rank test) and on the final water cue trial ($p = 0.000000006$, Wilcoxon signed-rank test), suggesting that cue value is updated without needing to experience the water cue-water pairing in the thirsty state.

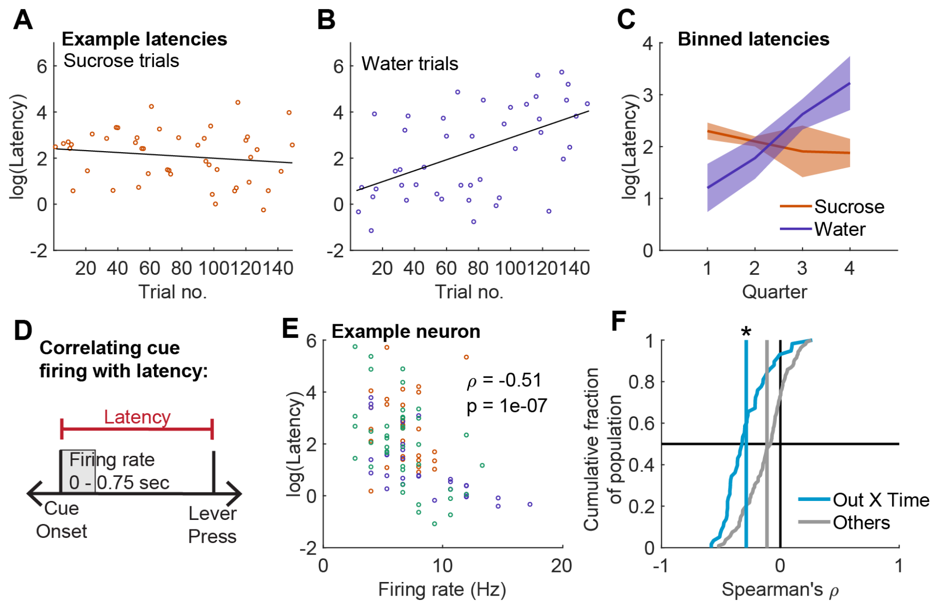

Figure S 4: Cue-evoked activity tracks reward-specific task performance.

(A) Latency to press lever (natural log-transformed) across all sucrose forced trials from an example Specific Cues session.

(B) As in (A), for water trials.

(C) Mean(+/−SEM) binned firing for Specific Cues sessions.

(D) Approach for calculating correlation between cue-evoked firing and latency to lever press.

(E) Correlation between firing rate for sucrose (orange), water (blue), and choice (green) trials and log(Latency) for an example Outcome X Time neuron.

(F) Distribution of correlation coefficients for Outcome X Time (blue) and other (gray) neurons. Mean of each group marked with vertical line. * indicates $p < 0.0000001$ for Wilcoxon rank-sum test comparing the groups.

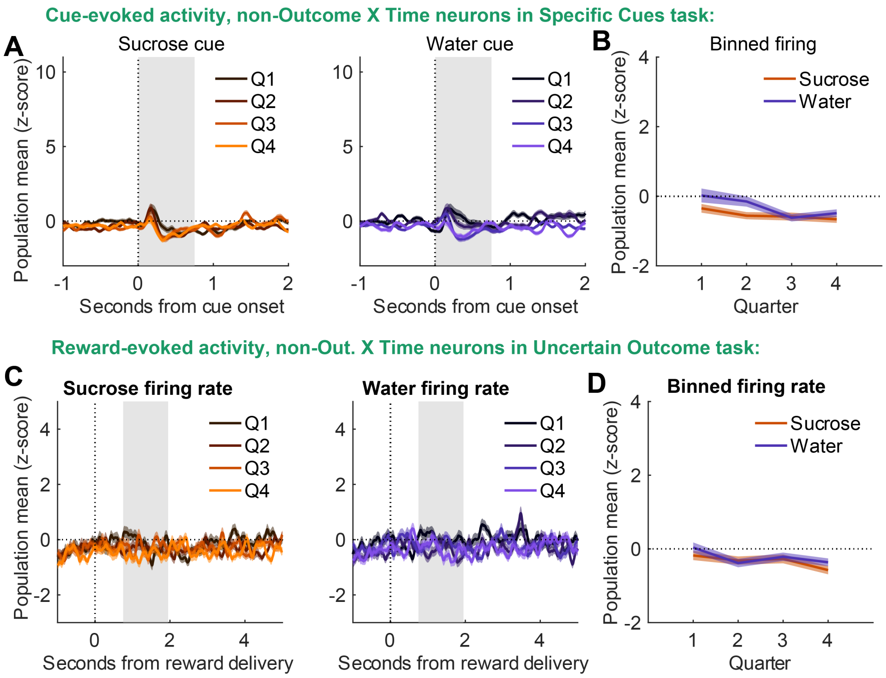

Figure S 5: Activity of non-Outcome X Time neurons.

(A) Mean(+/−SEM) sucrose (top) and water (bottom) cue-evoked firing for all non-Outcome X Time neurons from the Specific Cues task across the four quarters of trials. Gray shading indicates window for neural analysis.

(B) Mean(+/−SEM) binned firing for these neurons in this window.

(C) Mean(+/−SEM) sucrose- (left) and water-evoked (right) firing for all non-Outcome X Time neurons from the Uncertain Outcome task across the four quarters of trials. Gray shading indicates window for neural analysis.

(D) Mean(+/−SEM) binned firing for these neurons in this window.

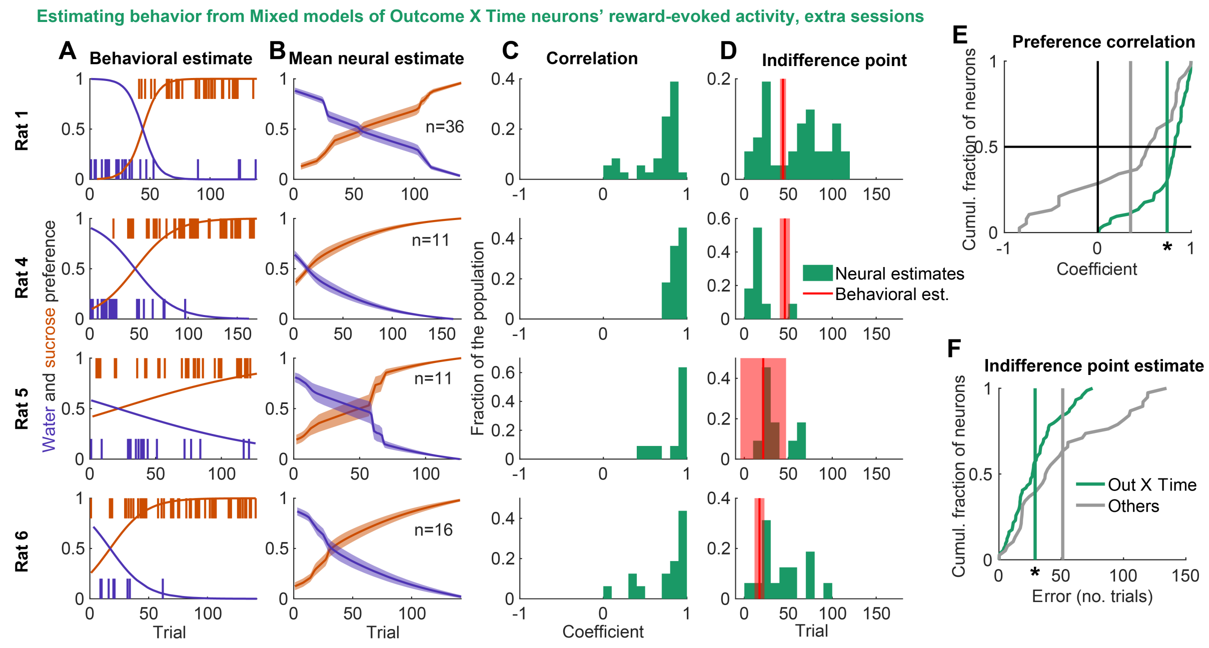

Figure S 6: Reward-evoked activity accurately predicts behavioral preference in additional Uncertain Outcome sessions.

(A) From four additional sessions not included in the main manuscript, the choices of the rats across the session and the preference estimated with a logistic function.

(B) The mean(+/−SEM) estimate of preference from fits of the Mixed model to the Outcome X Time neurons from these sessions.

(C) Correlation between neural estimate and behavioral estimate of preference for each Outcome X Time neuron from each of the sessions.

(D) Estimates of the indifference point (sucrose and water equally preferred) from the neural and behavioral (+/−SE) models.

(E) Across these Uncertain Outcome sessions, Outcome X Time neurons had preference estimates with higher correlations with the behavioral estimate than the remaining non-Outcome X Time neurons did ($p = 0.0003$, Wilcoxon rank-sum test).

(F) Outcome X Time neurons' estimates of indifference point were closer to the behavioral estimate than the remaining neurons ($p = 0.008$, Wilcoxon rank-sum test).

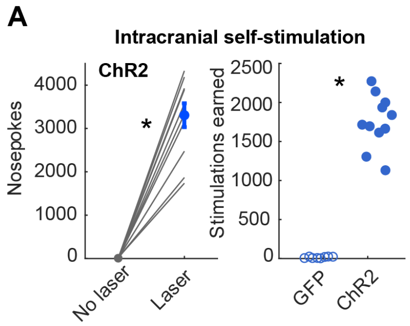

Figure S 7: Rats self-stimulate for optogenetic activation of VP.

(A) Intracranial self-stimulation (1s, 40Hz, unilateral) of VP via nosepoke port. Left: ChR2 rats made more nosepokes at the laser-paired port than on the unpaired port during the 1 hr session ($p < 0.001$, Wilcoxon signed-rank test). Right: ChR2 rats earned more stimulations during the 1 hr session than control rats ($p < 0.0001$, Wilcoxon rank-sum test).

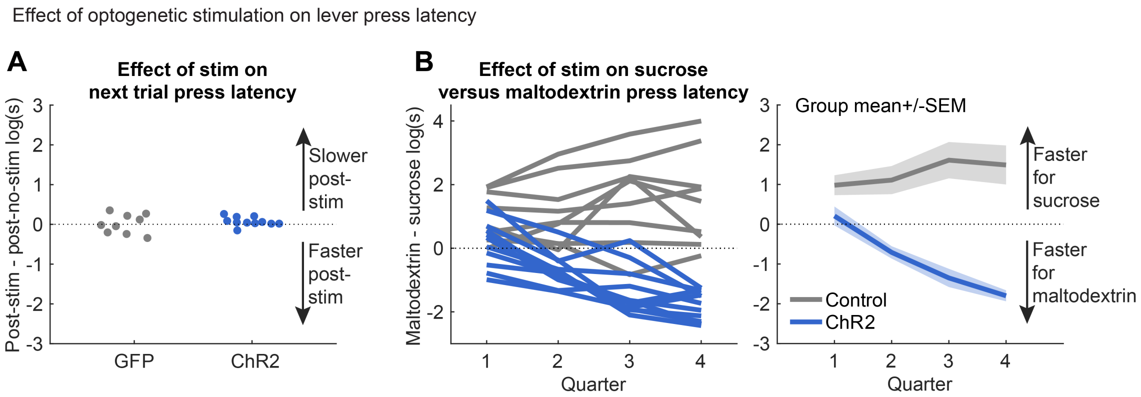

Figure S 8: Optogenetic stimulation on maltodextrin trials increases response vigor specifically for maltodextrin lever.

(A) In the test session from Fig. 4, optogenetic stimulation following receipt of maltodextrin did not impact (natural log-transformed) latency to press the lever on the following trial relative to the latency following trials without stimulation ($p = 0.59$, Wilcoxon rank-sum test comparing GFP and ChR2 groups).

(B) Difference between latency on sucrose and maltodextrin trials across the 4 quarters of trials for individual rats (left) and group averaged (right). While the difference in sucrose and maltodextrin press latency was similar for GFP and ChR2 groups in the first quarter ($p = 0.07$, Wilcoxon rank-sum test), the ChR2 group became quicker for maltodextrin relative to sucrose across the session (4th quarter versus 1st quarter, $p = 0.001$, Wilcoxon signed-rank test) while the control group remained similar (4th quarter versus 1st quarter, $p = 0.2$, Wilcoxon signed-rank test), resulting in significantly different difference scores for the two groups in the 4th quarter ($p = 0.0002$, Wilcoxon rank-sum test).
